# Combined Surgical and Orthodontic Treatment of Complex Odontoma in Growing Patients: Presentation of Two Cases

**DOI:** 10.3390/dj13020082

**Published:** 2025-02-14

**Authors:** Natalia Muczkowska, Ewa Czochrowska, Klaudia Masłowska, Andrzej Wojtowicz, Wojciech Popowski

**Affiliations:** 1 Department of Oral Surgery, Medical University of Warsaw, 02-097 Warsaw, Poland; natalia.muczkowska@wum.edu.pl (N.M.); klaudia.maslowska@wum.edu.pl (K.M.); andrzej.wojtowicz@wum.edu.pl (A.W.); 2 Department of Orthodontics, Medical University of Warsaw, 02-097 Warsaw, Poland; ewa.czochrowska@wum.edu.pl

**Keywords:** odontoma, odontogenic tumor, hamartoma, impacted tooth, orthodontic traction, self-eruption

## Abstract

**Background/Objectives:** Odontomas are the most common mixed odontogenic tumors and may cause impaction of adjacent teeth and masticatory disorders. Treatment of tooth impaction caused by the presence of odontomas is related to their stages of root development and their positions in the alveolar bone. The aim of this case report is to present the combined surgical and orthodontic treatment in growing patients with odontomas and to discuss the treatment outcomes. **Methods:** Two growing patients, an 8-year-old boy and a 17-year-old girl, with large odontomas in the posterior maxillary region of the maxilla were presented. The tumors were found during a radiological examination, and the first molars on the affected sides were impacted due to the presence of odontomas. **Results:** The treatment plans included the surgical removal of the tumors. In the case of the younger patient, the impacted developing molar erupted spontaneously in the oral cavity one year and two months after surgery. An orthodontic traction of the impacted molar was successfully applied in the older patient. **Conclusions:** The presence of a large odontoma in the posterior segments may lead to a displacement and impaction of neighboring molars and malformation of their roots. Spontaneous eruption of the affected molar can be expected if the tumor is diagnosed and removed early before its root formation is completed; otherwise, an orthodontic extrusion is needed. Interdisciplinary cooperation is important to diagnose and plan the dental treatment in young patients with odontomas.

## 1. Introduction

The term odontoma relates to mixed epithelial and mesenchymal tumor-like malformations derived from dental hard and soft tissues. Due to its limited and slow growth and well-differentiated tissue resembling tooth structures, an odontoma is considered as a hamartoma rather than a real tumor [1,2,3,4,5]. Depending on their internal composition, they are classified into complex odontomas (OCxs) composed of irregular masses of dentin, enamel, cementum, and compound odontomas (OCps) resembling tooth-like structures (“odontoids”), which additionally comprise the pulp tissue. These components are presented in different stages of histodifferentiation and morphodifferentiation and surrounded by the soft connective tissue capsule [1,2,3,4,6,7,8]. Due to their benign characteristics and low tendency of recurrence, they are considered to be hamartomas rather than neoplasms.

According to the fourth World Health Organization (WHO) Classification of Head and Neck Tumors (2017), odontomas are the most commonly occurring benign odontogenic lesion [5]. However, depending on the origin of the case series study, odontomas may be considered the most common or the second most common odontogenic tumor after ameloblastomas [9]. Buchner A. et al. evaluated 826 cases of odontomas in the American population, including both compound and complex incidences, which comprised 75.9% of all odontogenic tumors [3,4,5,10,11,12]. However, the study of Soluk-Tekkesin M. et al. in the Turkish population has confirmed that odontomas are the second most common odontogenic tumor (27.2%; *n* = 335, 27.2%) after ameloblastomas (29.7%; *n* = 366, 29.7%) [13,14,15]. The reasons for the reported variability in different populations might be associated with asymptomatic occurrence and incidental identification of odontomas during radiological examination [9,15].

Compound odontomas are often located in the anterior maxillary regions (74.6%), while in contrast, complex odontomas are frequently found in the posterior mandibular regions (68.2%). Usually, odontomas are asymptomatic, are diagnosed during routine radiological examination in the second decade of life, are rare in deciduous dentition, and have no sex predilection [2,5,9,13,16].

In clinical practice, odontomas frequently interfere with the eruption of permanent teeth, causing their impaction, malposition, or malformation, which cause malocclusion and esthetic problems if located in the anterior maxillary region. The delayed eruption of adjacent teeth is the most common clinical symptom. The most frequently impacted teeth are maxillary incisors and canines, whereas the mandibular permanent molars are affected less often. Other symptoms, such as dental and facial asymmetry, agenesis of permanent teeth, pain, or the presence of inflammation, are less common [2,3,13,17].

The recognition of a compound odontoma is confirmed by its tooth-like appearance of radiopaque well-defined lesions. The differential diagnosis of complex odontoma depends on different stages of its hard tissue mineralization, which may radiologically resemble different odontogenic tumors [13,18]. Also, it is reported that odontoma might occur as a component of a calcifying odontogenic cyst—approximately 20% of COCs are associated with odontoma [19]. Hence, the diagnosis of odontoma must be confirmed by histopathological examination [9,18,20,21].

The etiology of these lesions remains unknown. Odontoma has been related to many pathological factors, including an inflammatory process, hereditary anomalies such as Gardner’s syndrome [5,22], modifications of genes controlling the tooth development, or hyperactivity of odontoblasts [7,16,23,24,25,26]. It is also suggested that there is an association between trauma of deciduous teeth and odontomas [11,17,27].

The treatment of odontomas requires its complete surgical removal, along with the associated soft tissues of the fibrous capsule. Orthodontic traction of impacted teeth due to the presence of an odontoma may also be needed. The location and development stage of odontomas might have a significant effect on the adjacent tooth—if the lesion was extirpated early, the impacted tooth would normally complete its root formation and erupt spontaneously. If the location of odontoma is close to the adjacent tooth or teeth, and its atraumatic removal is not possible, extraction should be considered [28,29].

The aim of this case report is to present the combined surgical and orthodontic treatment in growing patients with odontomas and to discuss the treatment outcomes.

## 2. Case Reports

### 2.1. Case Report 1

A boy, 8 years and 10 months old, was referred by the family dentist to the Department of Oral Surgery, Medical University in Warsaw, due to an unerupted mandibular left first permanent molar in November 2019. He was generally healthy, without a history of a previous orthodontic treatment or trauma.

**Figure 1:**
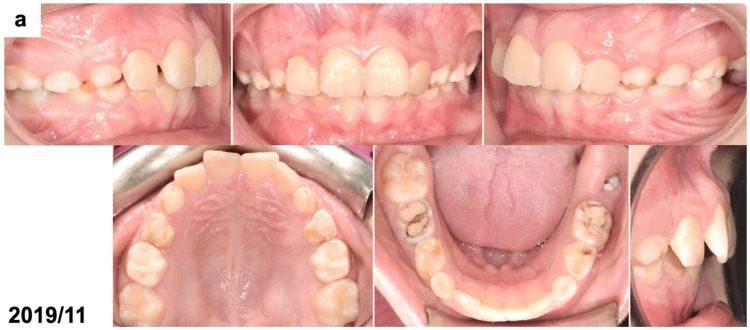

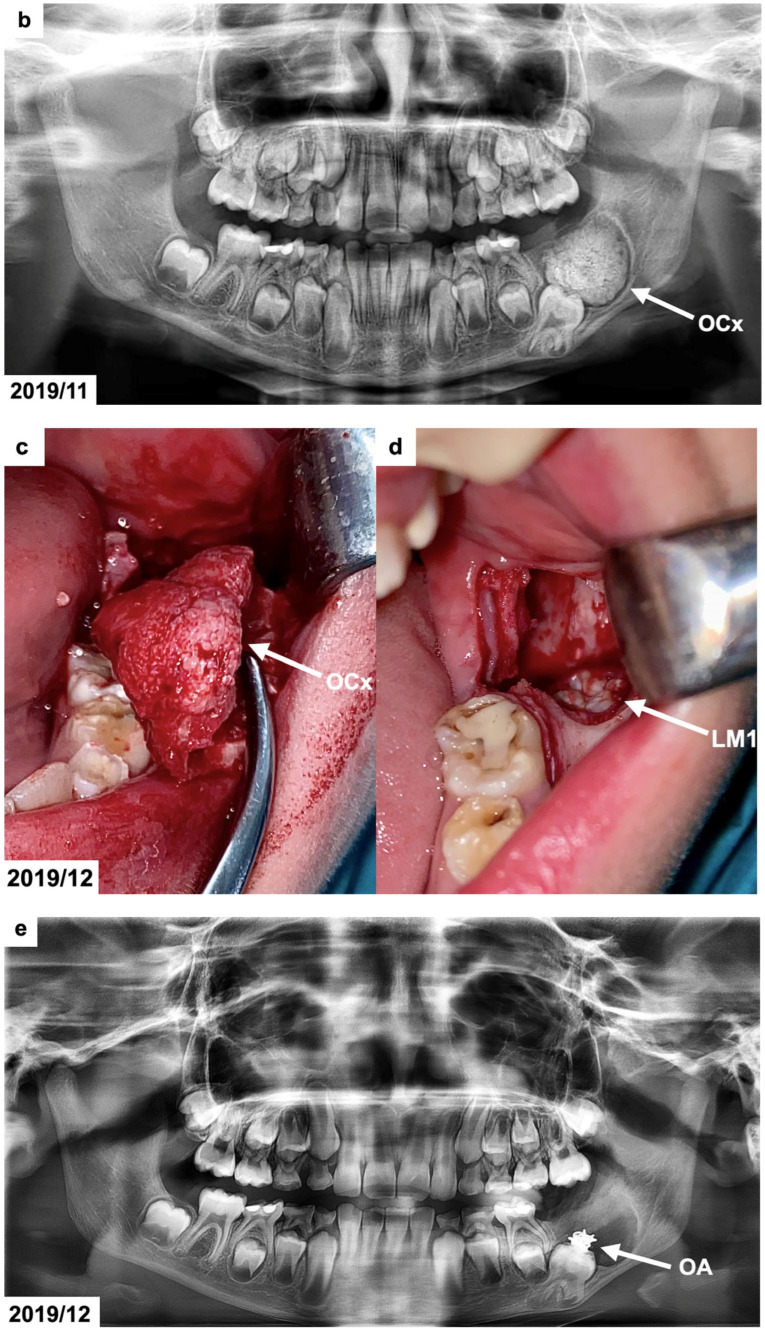

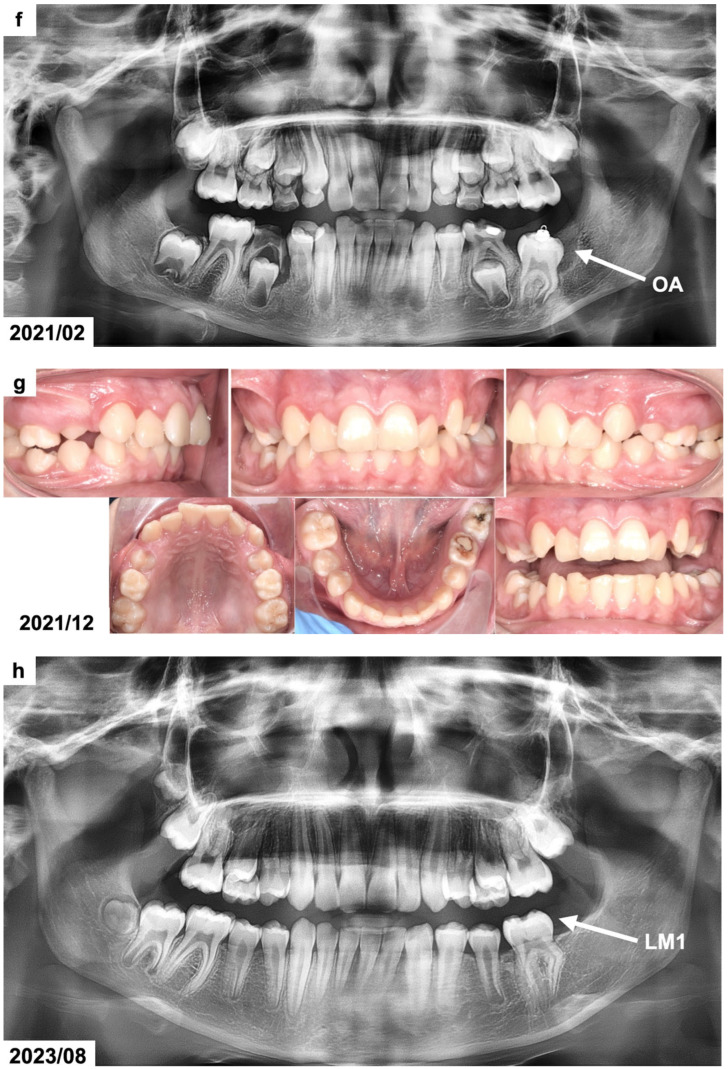

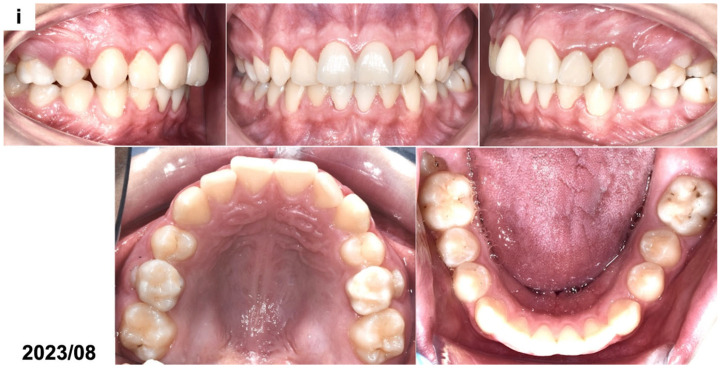
(**a**) Intraoral photographs before surgical and orthodontic treatment. The patient was 8 years and 10 months old. (**b**) Panoramic radiograph before the treatment, presenting the odontogenic tumor in the left mandible molar region (OCx = complex odontoma). The patient was 8 years and 10 months old. (**c**,**d**) Intraoral photographs taken during the surgical removal of the tumor exposing the occlusal surface of the permanent lower left first molar (OCx = complex odontoma, LM1 = lower left first permanent molar). The patient was 8 years and 11 months old. (**e**) Panoramic radiograph after the surgical treatment and bonding of the orthodontic attachment (OA = orthodontic attachment). The patient was 8 years and 11 months old. (**f**) Panoramic radiograph 1 year after the removal of the tumor showing the orthodontic attachment bonded to the occlusal surface of the permanent lower left first molar (OA = orthodontic attachment). The patient was 10 years and 1 month old. (**g**) Intraoral photographs of spontaneously erupted permanent lower left first molar after removal of the orthodontic attachment. The patient was 10 years and 11 months old. (**h**) Panoramic radiograph 4 years after the surgical and orthodontic treatment (LM1 = lower left first permanent molar). The patient was 12 years and 7 months old. (**i**) Intraoral photographs 4 years after the surgical and orthodontic treatment showing the erupted permanent lower left first molar and its position in the dental arch. The patient was 12 years and 7 months old.

The patient’s face was symmetrical and proportional, and the profile was convex, with a slightly retruded chin. The lips were competent, and no oral dysfunctions or parafunctions were present. The patient was at the early mixed dentition period with all incisors and first permanent molars erupted, except for the mandibular left first molar. All deciduous canines and molars were present. The oral hygiene was poor, and the deciduous molars had large fillings. An extraoral examination revealed Class II dental relations with an increased overjet and overbite and a small space deficiency in the upper anterior segment. A small part of the clinical crown of the mandibular left first molar was visible through the gingiva (Figure 1a). Orthodontic documentation, including extraoral and intraoral photographs, diagnostic models, and a cephalometric radiograph, was taken before the start of the treatment. An extraoral examination revealed an enlarged, non-painful lymph node, differentiated with bone swelling by the lesion. The intraoral examination showed poor oral hygiene, teeth with numerous fillings and active caries, pathological abrasion, and no signs of soft tissue inflammation; the dimensions of the alveolar process were unchanged.

The panoramic radiograph (Figure 1b), which was provided by the patient, indicated the presence of a calcified lesion with a radiolucent rim and an adjacent cortical bone layer with a diameter of approx. 3 cm at the distal area of the mandibular first permanent molar. To precisely define the size and position of the tumor, the patient was referred for cone-beam computed tomography (CBCT). The CBCT examination showed a focal area with a calcified mass with the radiodensity of hard dental tissues surrounded by a narrow radiolucent zone on the left side in the molar region of the mandible. The dimensions of the lesion were 25 × 20 × 17 mm. The radiological picture indicated the presence of a complex odontoma. This lesion extended to the lower border of the mandible. The position of the neighboring first permanent molar was altered by the tumor as the tooth was moved downward and mesially. The roots of the affected molar were still developing (app. 2/3 of the final root length). The upper part of the tumor was not covered by the alveolar bone by 20 × 6 mm.

The treatment plan included the surgical removal of the tumor—complex odontoma—and bonding of the orthodontic attachment for orthodontic extrusion of the impacted lower left first molar. The orthodontic extrusion was planned with the use of a lower removable appliance. This was related to the limited biomechanics in a young patient at this part of the oral cavity and because the cost of a removable appliance was refunded by the National Health System in Poland.

The surgery was performed under local anesthesia using lignocaine 2% with noradrenaline. In the retromolar area of the mandible, a triangular incision and a mucoperiosteal flap retraction were performed, exposing the rough surface of the tumor and the adjacent bone. A bony layer covering the lesion topically and buccally was removed using a round surgical bur with constant saline solution irrigation. After the tumor exposure, it was separated and removed entirely without damaging the unerupted first permanent molar (Figure 1c,d). The cavity was rinsed with a Metronidazole solution. The orthodontic attachment was bonded to the occlusal surface of the exposed tooth (Figure 1e). The flap was repositioned, and sutures were placed. The removed tumor was submitted for histopathological examination. Post-operatively, the patient was given an antibiotic (amoxicillin 500 mg/clavulanic acid 125 mg) every 12 h for 7 days. A follow-up visit was scheduled after 7 days of uneventful healing, and the sutures were removed. No post-operative complications were noted.

The H&E staining of decalcified section showed the composition of dentinal tissues arranged in a haphazard manner with irregular borders, confirming the clinical–radiographic diagnosis of complex odontoma.

The lower removable plate with an extension to attach the elastic from the button bonded on the exposed impacted molar was prepared in March 2020. However, due to the eruption of the COVID-19 pandemic, it was not delivered to the patient after surgery. The university clinic was only treating emergencies for the next few months, and the patient came back after 1 year, in February 2021. The patient reported a spontaneous eruption of the impacted maxillary. The occlusal surface of the lower left first permanent molar with a bonded button was visible during the clinical examination. The panoramic radiograph (Figure 1f) revealed a distinct occlusal movement of the impacted tooth and normal healing after the removal of the tumor. In December 2021, the mandibular left first permanent molar erupted in the oral cavity (Figure 1g). No pathologies were present on the panoramic radiograph except for the bending of the distal root of the affected molar. The patient has received an activator with an expansion screw to correct his Class II relations and maxillary crowding. In August 2023, the impacted molar tooth erupted into occlusion, and the root development was finished (Figure 1h,i). A marked dilaceration of its distal root could be noticed, which was caused by the presence of a tumor in proximity to the developing, neighboring first molar. The prognosis of the affected molar was estimated as favorable in the long term if good oral hygiene is maintained. A complete regeneration of the alveolar bone defect was also seen. No other permanent molars on the side of the tumor were present on the radiograph. Orthodontic treatment with a removable appliance is continued.

### 2.2. Case Report 2

A girl, 15 years and 10 months old, was referred by the family dentist for further diagnosis and treatment to the Department of the Oral Surgery, Medical University in Warsaw, due to the impaction of maxillary molars on the right side and the presence of an intraosseous lesion in the posterior maxillary segment in May 2015. The lesion was diagnosed on the panoramic radiograph, which was ordered by the referring dentist.

The patient was generally healthy, with no history of earlier orthodontic treatment or dental trauma. No discomfort or painful symptoms in the facial area or any TMJ signs or symptoms were reported. She had no complaints regarding her facial and dental esthetics and did not express a need for orthodontic treatment.

The face was symmetric and proportional, and the profile was straight. The intraoral examination revealed an absence of maxillary molars on the right side. The lymph nodes were not palpable. The oral hygiene was good, without any active caries or extensive restorations. The alveolar process in the right posterior region of the maxilla was enlarged, but it was not painful and without any signs of soft tissue inflammation.

The patient had Class I canine and molar dental relations without space problems in the dental arches. The overjet and overbite were normal. A posterior crossbite on the right side was diagnosed with a very slight midline deviation of the lower dental arch to the left side (1 mm). The upper midline coincided with the facial midline. No pathologies within hard or soft dental and periodontal tissues were seen.

**Figure 2:**
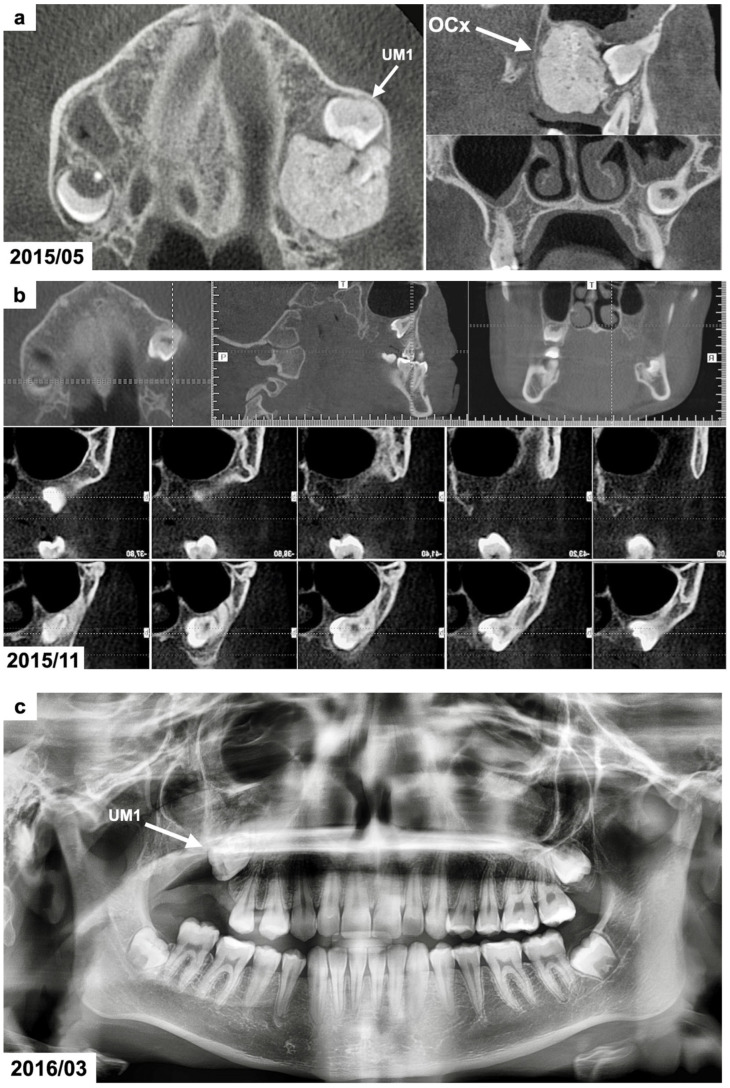

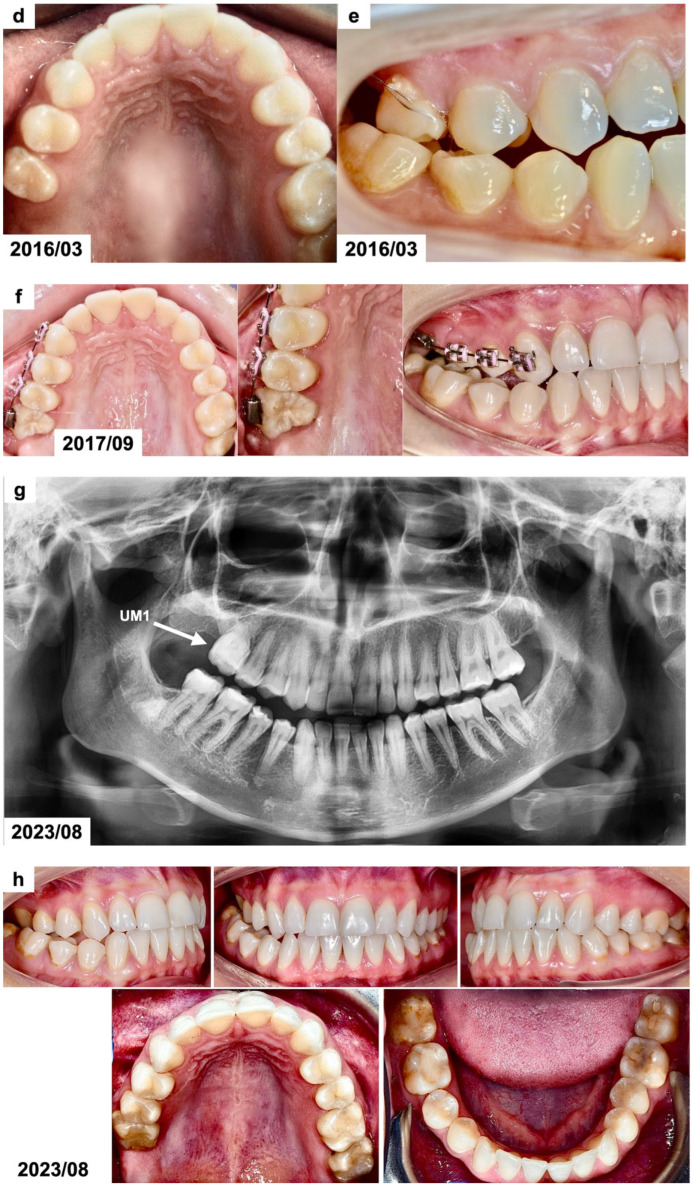

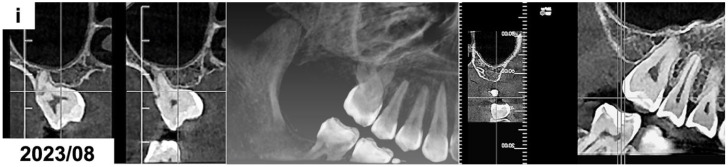
(**a**) CBCT examination scans before the treatment presenting the odontogenic tumor in the right maxillary molar region. The patient was 15 years and 10 months old. (**b**) CBCT examination scans after the surgical removal of the tumor presenting the malposition of the impacted permanent upper right first molar. The patient was 16 years and 4 months old. (**c**) Panoramic radiograph after the treatment, presenting the impacted maxillary. The patient was 16 years and 7 months old. (**d**,**e**) Intraoral photographs 10 months after the surgical treatment before the use of orthodontic traction with a segmented fixed appliance. The patient was 16 years and 7 months old. (**f**) Intraoral photographs 1 year and 4 months after the orthodontic traction of the permanent upper right first molar. The patient was 17 years and 10 months old. (**g**) Panoramic radiograph 6 years after the surgical and orthodontic treatment. The patient was 24 years and 1 month old. (**h**) Intraoral photographs 6 years after the surgical and orthodontic treatment showing the erupted permanent upper right first molar and its position in the dental arch. The patient was 24 years and 1 month old. (**i**) CBCT examination scans 6 years after the surgical and orthodontic treatment presenting the erupted permanent upper right first molar and the absence of the alveolar bone at the site of the tumor removal. The patient was 24 years and 1 month old.

The panoramic radiograph (provided by the patient) revealed the presence of a radiodense lesion surrounded by a narrow radiolucent zone at the posterior area of the right maxilla, which was the suspected reason for the impaction of the right maxillary first permanent molar (Figure 2a).

Cone-beam computed tomography (CBCT) was scheduled to determine the dimensions and position of the tumor. The CBCT examination showed a focal area with spherical radiopaque mass resembling the radiodensity of irregularly composed hard dental tissues/dentin and enamel tissues surrounded by a thin radiotransparent rim in the right maxillary molar region (Figure 2b). The dimensions of the lesion were 16 × 18 × 20 millimeters. The roots of the maxillary first permanent molar were fully developed. The tooth was impacted at distoangular position and its crown occlusally contacted with the tumor. The maxillary right second and third molars were absent. Based on the CBCT examination, the presence of a complex odontoma was suspected.

The treatment plan included surgical removal of the tumor and a combined surgical and orthodontic extrusion of the impacted maxillary molar. Bonding of an orthodontic button with a soft ligature for orthodontic traction was planned during the surgical removal of the tumor. The patient was not interested in the comprehensive orthodontic treatment to correct the posterior crossbite. Therefore, a segmented fixed orthodontic appliance in the maxillary posterior segment on the right side was planned, including the canine and both premolars. The orthodontic traction of the impacted molar was planned after the surgical removal of the tumor and lack of a spontaneous eruption. The patient was negative regarding the use of any temporary skeletal anchorage devices (TADs) from the start of orthodontic traction.

The surgery was performed under local anesthesia using lignocaine 2% with noradrenaline. A trapezoidal incision was made in the right maxillary molar area, followed by a mucoperiosteal flap exposing the tumor surface. Cortical bone covering the lesion buccally was removed using a round surgical bur with saline irrigation. After the tumor exposure, it was possible to remove the tumor entirely without damaging the underlying first molar. The cavity was curetted and rinsed with a Metronidazole solution. An orthodontic attachment was bonded to the crown of the impacted first molar with a metal ligature attached to the second premolar. The flap was repositioned, and the sutures were placed. The removed tumor was sent for a histopathological examination. Post-operatively, the patient was given an antibiotic (amoxicillin with clavulanic acid; 0.625 g) every 12 h for 7 days. The healing was uneventful, and after 7 days, the sutures were removed.

The result of a histopathological examination with H&E staining revealed the presence of mature tubular dentin with the spaces containing small amounts of enamel matrix or immature enamel—the mature enamel was removed during the decalcification process. Therefore, based on the clinical, radiological, and histological examinations, the diagnosis of a complex odontoma was confirmed. The impacted maxillary molar was left for observation, but no signs of its spontaneous eruption were seen for 10 months (Figure 2c–e). Therefore, an orthodontic traction with a segmented fixed appliance was planned (0.022 slot, American Orthodontics, Mini Master series). Orthodontic brackets were bonded on the maxillary canine and premolars on the right side. The teeth were aligned after 3 months, and a stainless-steel rectangular wire (0.017 × 0.022 SS) was placed. The impacted tooth was attached to the appliance using an elastomeric Powerchain attached to the metal ligature. The Powerchain was scheduled for changing every 5–6 weeks. The impacted molar was successfully extruded in the oral cavity after 1 year and 4 months (Figure 2f); however, the patient missed some appointments. The patient refused to continue her orthodontic treatment to correct the posterior crossbite after the extrusion of the impacted molar. The orthodontic appliance was debonded, and no retention was used. The patient missed scheduled follow-up appointments and presented for re-evaluation 5 years after finishing the orthodontic traction. The maxillary right first molar was present in the oral cavity without any signs of pathology (Figure 2g,h). The cone-beam computed tomography revealed the absence of the alveolar bone at the site of the tumor removal. It was recommended to perform bone augmentation procedures before further implantation (Figure 2i).

## 3. Discussion

Odontomas are the most common intraosseous odontogenic tumors, usually diagnosed in the first two decades of life. They may lead to the displacement of adjacent teeth, delayed tooth eruption, or the impaction of permanent teeth and occlusal disturbances [18,28,30]. In both described cases, the odontomas were in the posterior parts of the maxilla or mandible and caused the absence and impaction of neighboring permanent teeth. The patients were growing patients at different stages of tooth development, and therefore, an absence of a single posterior tooth was not that easily recognized by the patients or their parents. However, in Case 2, earlier detection of a tumor could be expected during a routine dental examination. The lesions were generally asymptomatic, with slight enlargement of the alveolar process at the posterior parts of the dental arches. In the first case, the lesion was detected on a panoramic radiograph taken during a routine orthodontic consultation, while in the second case, the clinical dental examination revealed an absence of a maxillary first molar. The panoramic radiograph has confirmed the presence of an odontoma, which had caused the impaction of the neighboring molars. In both cases, the tumors were surgically removed, and an orthodontic treatment was planned to extrude the impacted molars that were left after the surgery. It is important to plan an interdisciplinary treatment in growing patients with odontomas. Pediatric dentists might be the first dental specialists to diagnose the presence of a tumor in a young patient, and orthodontists should be involved in the treatment planning after the odontoma removal.

It is important to differentiate complex odontomas from other tumors, including ossifying fibromas (OFs), osteomas, cemento-osseus dysplasias (CODs), ameloblastic fibromas (AFs), ameloblastic fibro-odontomas (AFOs), ameloblastic fibro-odontomas (AFDs), and calcifying odontogenic cysts (COCs), using both radiological and histological features that are specific to confirm the explicit diagnosis [7,16,18,21]. Tumors previously identified as AFDs and AFOs contain a calcified matrix of hard dental tissues that resembles an odontoma and a soft tissue component resembling an ameloblastic fibroma. The features of AFO and AFD appear to be intermediate between an AF and an odontoma. Currently, AFDs and AFOs are classified as developing odontomas, despite the presence of BRAF p.V600E mutations that are characteristic of AFs, in contrast to OCxs, which lack these mutations. Additionally, cases of AFO/AFD with locally aggressive biological behavior suggest their neoplastic rather than hamartomous character. Nonetheless, further molecular research is expected to elucidate whether AFDs and AFOs are separate lesions or a combination of developing odontomas and ameloblastic fibromas [5,9,14]. In the presented cases, the histological examination of both tumors performed after their surgical removal confirmed the diagnosis of complex odontoma.

The etiology of the odontoma is yet uncertain. Many aspects are indicated to induce the development of odontomas, such as inflammatory, traumatic, or genetic factors. In the presented cases, no clear etiological factor, such as a previous dental pathology or trauma, was reported. Also, a family history of tooth impaction was negative. The patients did not report any pain or functional problems related to the presence of the odontoma. Both patients were lacking second and third permanent molars on the tumor site, which was probably caused by the presence of the odontomas [25,31]. Liu J.K. et al. (1997) presented a case report of a 12-year-old boy who suffered from impaction of the mandibular first permanent molar due to the presence of an odontoma. The absence of the mandibular second and third permanent molars was also reported. Liu J.K. et al. (1997) concluded that the odontoma might have been caused by disturbances in the development of these teeth [31].

According to the literature, the treatment of choice is the surgical removal of the odontoma with the preservation of the impacted tooth, which is usually separated by a septum of bone [2,24,32]. It aims at the spontaneous eruption of the affected impacted tooth, especially if it is performed before the root development is completed. There are various factors, including the position of the impacted tooth in the alveolar process, its morphology and the stage of root development, and space conditions, that should be considered when planning the postsurgical treatment [11,17,28,30,32]. The treatment of odontoma-related tooth impaction includes the removal of the lesion with a surgical exposure of the impacted tooth and usually the bonding of an orthodontic attachment for extrusion. An important factor is the stage of root development of the impacted tooth, as teeth with developing roots are more likely to have a spontaneous eruption after surgery. Because of that, an early diagnosis of the lesion that impedes a normal tooth eruption is important. The family dentists clinically and radiologically diagnosed eruption disturbances in the presented patients and referred them to the specialist in oral surgery for further treatment. Since odontomas are usually separated from adjacent teeth by a bony septum, tooth extraction should only be considered if there is no possibility of tumor removal without damaging the adjacent tooth [24,25,32].

In Case 1, the tumor was diagnosed early, during the mixed dentition period, when the root formation of an impacted molar was not completed. In such cases, the surgical removal of the tumor enabled the spontaneous eruption of the permanent tooth without a need for orthodontic traction. The orthodontic observation should be continued until the impacted tooth has reached occlusion [3,4,8,11,13,29,30]. The study by Hidalgo-Sánchez O. et al. analyzed 77 cases of odontomas management, and in all cases, the treatment consisted of the surgical removal of the tumor, and the necessity for posterior orthodontic treatment was mentioned only in 7 cases [2]. However, the study of Isola G. et al. reported that in their study sample, only 4 teeth erupted spontaneously after surgery, and 29 teeth were completely aligned through orthodontic–surgical treatment during a follow-up period of 15 years [13]. The possibility of the spontaneous eruption of an impacted tooth after odontoma removal is related to the stage of its root development, its position in the alveolar bone, and its relation to the neighboring teeth [3,16,17,18,24,28,29,30,31,32,33].

In Case 2, the tumor was diagnosed in full permanent dentition after the completion of the root development of the first molar. The impacted tooth did not erupt spontaneously, and therefore, orthodontic traction with a fixed appliance was necessary to align the tooth in the dental arch. Early diagnosis of such tumors is, therefore, important as their early removal could minimize the need for an orthodontic traction. The presence of orthodontic appliances may lead to enamel demineralization, risk for root resorption, and other iatrogenic effects, such as soft tissue irritations or allergies. The extrusion of the terminal molar in the dental arch also provides uncertain results due to the need for anchorage control. In the described case, the impacted tooth was successfully extruded without a need for additional anchorage reinforcement, including temporary skeletal devices (TADs). Also, the insertion of TADs after the removal of a massive tumor located in the alveolar process might be difficult, and other sites of the dental arch should be considered, such as palatal TADs.

The recurrence of odontomas is rare, and the prognosis is extremely good [5,7,8,10]. Regarding the research of Hisatomi M. et al., odontomas that were not associated with impacted teeth were left for observation for several years, and they did not change in terms of location, size, or radiographic findings during the follow-up period. Due to their limited growth and lack of cell proliferation or malignancy, the recurrence risk of odontomas is very low. The traditional surgical treatment, with histopathological examination to confirm the diagnosis, followed by orthodontic traction of impacted teeth, if necessary, is the best method for their management [5,25,28,29,30,34,35,36,37]. In both cases, no recurrence of the tumor was observed during the treatment.

## 4. Conclusions

The presence of large odontomas in the posterior segments leads to the displacement and impaction of neighboring molars. Spontaneous eruption of the affected molar can be expected if the tumor is diagnosed and removed early, before its root formation is completed. Surgical removal of the tumor should aim at preservation of the adjacent teeth, and orthodontic extrusion is often needed to extrude impacted molars. Interdisciplinary cooperation is important to diagnose and plan the dental treatment in young patients with odontomas.

## Data Availability

The data are available upon reasonable request from the corresponding authors.

## Abbreviations

OCx—complex odontoma, OCp—compound odontoma, OF—ossifying fibroma, COD—cemento-osseus dysplasia, AF—ameloblastic fibroma, AFO—ameloblastic fibro-odontoma, AFD—ameloblastic fibro-odontoma, COC—calcifying odontogenic cyst, CBCT—cone-beam computed tomography, TMJ—temporomandibular joint, TADs—temporary skeletal devices.
